# La_2_Pd_3_Ge_5_ and Nd_2_Pd_3_Ge_5_ Compounds: Chemical Bonding and Physical Properties

**DOI:** 10.1021/acs.inorgchem.0c03744

**Published:** 2021-02-11

**Authors:** Riccardo Freccero, Serena De Negri, Gerda Rogl, Georg Binder, Herwig Michor, Peter F. Rogl, Adriana Saccone, Pavlo Solokha

**Affiliations:** † Università degli Studi di Genova, Dipartimento di Chimica e Chimica Industriale, Via Dodecaneso 31, I-16146 Genova, Italy; ‡ Institute of Materials Chemistry, University of Vienna, Währingerstraße 42, A-1090 Vienna, Austria; § Institute of Solid State Physics, TU Wien, Wiedner Hauptstraße, 8-10, A-1040 Wien, Austria

## Abstract

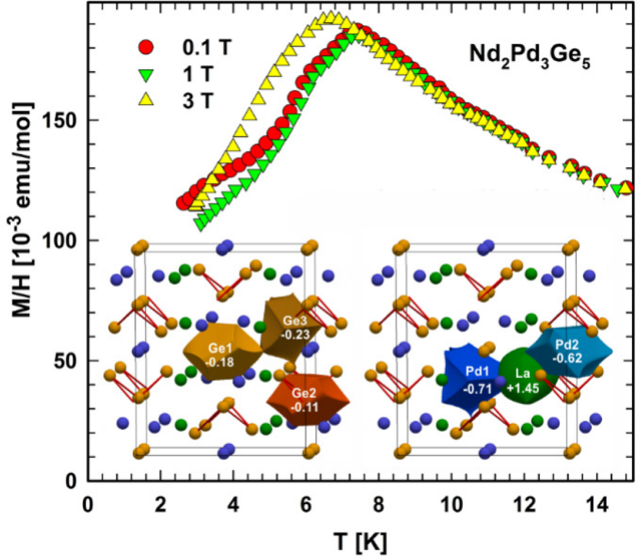

The two La_2_Pd_3_Ge_5_ and Nd_2_Pd_3_Ge_5_ compounds, crystallizing in the *oI*40-U_2_Co_3_Ge_5_ crystal structure, were targeted for analysis of their chemical bonding and physical properties. The compounds of interest were obtained by arc melting and characterized by differential thermal analysis, scanning electron microscopy, and X-ray diffraction both on powder and on a single crystal (for the La analogue), to ensure the high quality of the samples and accurate crystallographic data. Chemical bonding was studied by analyzing the electronic structure and effective QTAIM charges of La_2_Pd_3_Ge_5_. A significant charge transfer mainly occurs from La to Pd so that Ge species assume tiny negative charges. This result, together with the -(I)COHP analysis, suggests that, in addition to the expected homopolar Ge bonds within zigzag chains, heteropolar interactions between Ge and the surrounding La and Pd occur with multicenter character. Covalent La–Pd interactions increase the complexity of chemical bonding, which could not be adequately described by the simplified, formally obeyed, Zintl–Klemm scheme. Electric resistivity, specific heat, magnetization, and magnetic susceptibility as a function of temperature indicate for both compounds a metallic-like behavior. For Nd_2_Pd_3_Ge_5_, two low-temperature phase transitions are detected, leading to an antiferromagnetic ground state.

## Abstract 2

The chemical bonding and physical properties of the two isotypic *R*_2_Pd_3_Ge_5_ (R = La and Nd) intermetallics are presented. La_2_Pd_3_Ge_5_ shows polar Ge−Pd/La multicenter interactions in addition to covalent Ge−Ge bonds. The bonding scenario is further complicated by the fact that Pd and La are also covalently interacting. For Nd_2_Pd_3_Ge_5_, an antiferromagnetic ground state is established after a long-range magnetic ordering (at ∼7.5 K) followed by a spin-reorientation transition (at ∼6.2 K).

## 1 Introduction

Combination of Ge with more electropositive metals leads to a great variety of compounds, containing different Ge-based fragments, the extension and shape of which depend both on the composition and on the nature of the other constituent(s).

When the electronegativity difference is remarkable (for example, with alkali metals), an almost complete charge transfer can be assumed and covalently bonded anionic Ge fragments form. For the Ge-richest compositions, the so-called “intermetallic clathrates” feature infinite frameworks composed by four-bonded (4*b*)Ge and three-bonded (3*b*)Ge forming large cages hosting the alkali atoms.^1^ With a decrease in germanium content, discrete and stable anionic Ge clusters form, which can even be extracted from the solid and functionalized with various molecular ligands, as, for example, in K_4_Ge_9_.^2,3^ Chemical bonding in these compounds is frequently approached by applying the Zintl–Klemm concept. When the electronegativity difference is decreased by substituting an alkali with an alkaline-earth metal, the reduced charge transfer leads to covalent interactions among cations and the anionic partial structures. In these cases, although the Zintl–Klemm rules are frequently fulfilled, they are, indeed, not sufficient to account for all of the different kinds of interactions taking place.^4^ Modern quantum chemical approaches based on QTAIM^5^ and electron localizability ELI-D^6−8^ have been applied to achieve more insights into chemical bonding features.^4,9^

Another wide group of germanides forms when the electropositive counterpart is represented by a rare-earth metal (*R*) and furthermore by adding a third element, for example, of the *d*-block.^10^ In these compounds, due to a less pronounced charge transfer, many different kinds of Ge fragments are observed, such as (3*b*)Ge infinite networks (as in *R*Ge_2_ and their derivatives)^10^ or corrugated layers (as in *R*_2_*M*Ge_6_, where *M* = another metal),^11−13^ (2*b*)Ge zigzag chains with different topologies (as in *R*Al_1–*x*_Ge_2_,^14^*R*_2_*M*Ge_6_,^11−13^*R*_2_*M*_3_Ge_5_,^15−18^ and LuGe^19^), and simpler isolated fragments like *cis*-Ge_4_ (as in Lu_5_Pd_4_Ge_8_^20^) and Ge–Ge dumbbells (as in La_4_Mg_5_Ge_6_,^21^*R*_2_*M*Ge_2_,^22,23^ Lu_5_Pd_4_Ge_8_, and Lu_3_Pd_3_Ge_4_^20^). The occurrence of these Ge motifs with peculiar geometries mainly depends on two chemical factors: the composition and the nature of Ge–metal and metal–metal interactions. In fact, a higher content of electropositive metals causes a larger number of valence electrons formally transferred to Ge, so simpler Ge fragments, characterized by a reduced number of Ge–Ge bonds, occur. The geometry of the resulting fragments is strongly dependent on the presence of stabilizing bonding interactions, more complex than the ionic–covalent ones described by the Zintl model. Therefore, the application of the Zintl–Klemm concept to these compounds usually fails.

Recently, chemical bonding in the Ge-rich *R*_2_*M*Ge_6_ compounds was studied by COHP/ICOHP for La_2_ZnGe_6_^11^ and, at a second stage, for La_2_*M*Ge_6_ (*M* = Li, Mg, Al, Cu, Zn, Pd, or Ag)^13^ and Y_2_PdGe_6_ applying the QTAIM and ELI-D position-space techniques. In all cases, in addition to the expected Ge–Ge covalent bonds, the Ge–La/*M* (*M* = Al, Cu, Zn, Pd, or Ag) interactions were described as polar–covalent. The increased complexity of the bonding situation when moving from the main group to the transition metal analogues was also revealed by the presence of La–*M* heteropolar interactions, traceable when deeply analyzed by means of the ELI-D fine structure based on its relative Laplacian.

Another large family of ternary germanides with a lower Ge content is that with a general formula of *R*_2_*M*_3_Ge_5_. Numerous studies previously examined the crystal structure and physical properties of these intermetallics,^15−18,24^ also because many of them form easily, and high-yield samples can be obtained by simple arc or induction melting with no further thermal treatments. Nevertheless, chemical bonding studies of these germanides are almost absent. To fill this gap, chemical bonding in La_2_Pd_3_Ge_5_ was studied, and the results are reported in this work together with some measurements of physical properties.

## 2 Experimental Section

### 2.1 Sample Preparation and Scanning Electron Microscopy (SEM)/Energy Dispersive X-ray Spectroscopy (EDXS) Characterization

Samples with a nominal composition of *R*_20.0_Pd_30.0_Ge_50.0_ (*R* = La or Nd) were prepared starting from stoichiometric amounts of the pure constituents, all with nominal purities of >99.9 mass %. Elemental lanthanum or neodymium (both supplied by Newmet Koch, Waltham Abbey, England) and palladium and germanium (supplied by MaTecK, Jülich, Germany) were arc melted on a water-cooled copper hearth with a tungsten electrode under an Ar atmosphere, yielding very brittle ingots of ∼0.8 g that proved to be stable in air. Mass losses were <0.5%.

For metallographic analysis, some fragments of each alloy were embedded in a phenolic resin with graphite filler, employing an automatic hot compression mounting press (Opal 410, ATM GmbH). The sample surfaces were smoothed with an automatic grinding and polishing machine (Saphir 520, ATM GmbH). A multistep grinding and polishing procedure was performed using SiC papers, with grain sizes from 600 to 1200 mesh with running water as the lubricant, and finally diamond pastes from 6 to 1 μm with an alcohol-based lubricant. Petroleum ether was employed after each polishing step to clean samples in an ultrasonic bath for a few minutes.

To examine microstructures and measure phase compositions, a scanning electron microscope (Zeiss Evo 40, Carl Zeiss SMT Ltd., Cambridge, England) was used, equipped with an energy dispersive X-ray (EDX) spectroscope from Oxford Instruments (INCA X-ACT). The compositional analysis was performed on the basis of the characteristic X-ray intensities of each element. A Co standard was used for calibration. The error in the measured EDXS composition is ∼0.5 atom % for each element.

### 2.2 X-ray Diffraction Measurements (XRD) and Crystal Structure Determination

A single crystal of La_2_Pd_3_Ge_5_ was selected from the crushed alloy. Its shape and composition were checked by SEM-EDXS prior to X-ray analysis (see the Supporting Information).

The data set was obtained in a routine fashion under ambient conditions on a Bruker Kappa APEXII diffractometer operating in the ω-scan mode, equipped with a CCD area detector and graphite monochromatized Mo Kα (λ = 0.071073 Å) radiation. The crystal, glued on a glass fiber, was mounted on the goniometric head, and intensity data were collected over the reciprocal space up to ∼30° in θ with exposures of 30 s per frame. Semiempirical absorption corrections based on a multipolar spherical harmonic expansion of equivalent intensities were applied to all data by the SADABS software.^25^

The structural model was easily found by direct methods and successively refined using full-matrix least-squares methods with the SHELX-14 package^26^ showing excellent residuals. The corresponding CIF file has been deposited at the Cambridge Structural Database as entry 1917376. Selected crystallographic data and structure refinement parameters are listed in Tables S1 and S2.

La_2_Pd_3_Ge_5_ is isostructural with other orthorhombic *R*_2_Pd_3_Ge_5_ compounds: space group *Ibam*, *oI*40-U_2_Co_3_Ge_5_, *a* = 10.1914(6) Å, *b* = 12.2082(7) Å, *c* = 6.1901(4) Å.

X-ray powder diffraction (XRPD) analyses were conducted on samples by means of a Philips X’Pert MPD diffractometer with a θ:2θ Bragg–Brentano geometry (Cu Kα radiation, λ = 1.5406 Å, graphite crystal monochromator, scintillation detector, step mode of scanning). Powder patterns were collected in the 2θ range of 10–100°, with a scanning step of 0.02° and a time per step of 15 s. Lattice parameters of the studied phases, refined by a least-squares routine, match well with those obtained by single-crystal analyses.^15^

### 2.3 Differential Thermal Analysis (DTA)

The melting temperature (*T*_m_) of the title compounds was measured by differential thermal analysis (DTA) using a LABSYS EVO instrument (SETARAM Instrumentation, Caluire, France), equipped with type S (Pt-PtRh 10%) thermocouples. Measurements were conducted in the temperature range of 25–1200 °C with a heating/cooling rate of 5 °C/min under an Ar flow (20 mL/min). Custom-made Ta crucibles were used as both sample containers (∼200 mg) and references. The loaded crucibles were arc-sealed under an Ar atmosphere after being cooled with liquid nitrogen to avoid undesired reactions. The recorded thermograms were evaluated and analyzed with the aid of Calisto, supplied by SETARAM. To calibrate the equipment, the measured melting temperatures of standard materials (Zn, Ge, and Cu) and their tabulated values^27^ were employed. Temperatures were defined from the onset point of the heating curve peaks, determined through an extrapolation procedure.

### 2.4 Magnetic, Specific Heat, and Electric Resistivity Measurements

Temperature- and field-dependent magnetization and dc magnetic susceptibility measurements were performed in the temperature and field intervals of 3–300 K and 0–6 T, respectively, with a cryogenic SQUID magnetometer. Temperature-dependent ac susceptibility measurements were carried out from 4.2 to 150 K with a revised Lakeshore 7000 AC susceptometer, applying an ac field with a root-mean-square amplitude of 400 A/m and a frequency of 400 Hz. Specific heat measurements on samples of ∼60 mg were carried out in two different ways: (i) by means of a homemade quasi-adiabatic step heating system in the temperature range of 2.2–15 K and (ii) with a Quantum Design PPMS relaxation-type calorimeter in the extended interval of 2–280 K. For the measurements of the electrical resistivity, a common four-probe ac technique with spot-welded gold contacts on a bar-shaped sample in the temperature range of 2–300 K was applied. In the temperature range of 4.2–10 K, the measurement was performed in steps of 0.05 K.

### 2.5 Computational Techniques

The electronic structure of La_2_Pd_3_Ge_5_ was studied with TB-LMTO-ASA version 4.7c,^28^ employing an exchange and correlation potential, according to Barth and Hedin,^29^ within the local density approximation (LDA). For this purpose, the experimental structural data presented in this work were used. The addition of empty spheres, to meet the minimum overlapping criterion, was not necessary, yielding the following atomic sphere radii: *r*(La) = 2.156 Å, *r*(Ge1) = 1.558 Å, *r*(Ge2) = 1.518 Å, *r*(Ge3) = 1.464 Å, *r*(Pd1) = 1.398 Å, and *r*(Pd2) = 1.507 Å. The self-consistent calculation was performed with a basis set that included La-6*s*/(6*p*)/5*d*/4*f*, Ge-4*s*/4*p*/(4*d*), and Pd-5*s*/5*p*/4*d*/(4*f*); parentheses indicate orbitals treated according to a downfolding procedure. The Brillouin zone was integrated with the tetrahedron method using 280 irreducible *k*-points from a total of 1728.

Chemical bonding was studied on the basis of crystal orbital Hamilton populations (COHPs)^30^ and the corresponding integrated values up to the Fermi level (ICOHP). Plots of densities of states (DOS) and COHP curves were generated using wxDragon,^31^ setting the Fermi energy at 0 eV as a reference point. The electron density (ED) was calculated on an equidistant grid of ∼0.05 Bohr using an implemented module^32^ within the FPLO software.^33^ For the sake of consistency, the same exchange and correlation potential applied in the previous calculations was employed sampling the Brillouin zone with an 8 × 8 × 8 *k*-point mesh. The ED was analyzed according to Bader’s quantum theory of atoms in molecules (QTAIM)^5^ using DGrid.^34^ As a result, the crystal space is separated into non-overlapping and space-filling regions called atomic basins. The integration of the electron density within QTAIM basins yields their average electronic populations; their subtraction from atomic numbers gives the effective charges. The ED and QTAIM atomic basins were visualized within the unit cell using ParaView.^35,36^

To thoroughly investigate the role of Pd, a comparative chemical bonding analysis was conducted considering the hypothetical “La_2_Mg_3_Ge_5_”. Its structure was simply generated by introducing Mg into Pd sites and geometrically optimized with the all-electron DFT-based FHI-aims package.^37,38^ To validate this computational procedure, also the La_2_Pd_3_Ge_5_ structure was relaxed under the same setup: Perdew and Zunger (LDA)^39^ exchange and correlation potential, (4 4 8) *k*-point mesh for Brillouin zone sampling, and predefined default “light” basis set for all atomic species. In addition, scalar relativistic effects were included within the “atomic ZORA” approximation; a Gaussian smearing of 0.01 eV was set up. The relaxed La_2_Pd_3_Ge_5_ unit cell parameters are in very good agreement with the experimental ones (see the Supporting Information).

Partial (*p*)DOS and COHP curves for selected interactions and ICOHP were calculated for relaxed La_2_Mg_3_Ge_5_ with the TB-LMTO-ASA (same setup as for La_2_Pd_3_Ge_5_). More details about the latter calculation and structural data of the relaxed La_2_Mg_3_Ge_5_ compound are reported in the Supporting Information.

## 3 Results and Discussion

The quality of the prepared samples was carefully checked by SEM-EDXS and XRPD (see the Supporting Information). Both are almost single-phase *R*_2_Pd_3_Ge_5_; a small amount of the eutectic (Ge+PdGe) mixture can be detected only by electron microscopy. The average microprobe compositions of the title phases, listed in Table 1, are in good agreement with the 2:3:5 stoichiometry. Lattice parameters calculated from powder and single-crystal diffraction data match well with each other (see Table 1) and fit in the trend of the cell volume as a function of the *R*^3+^ ionic radius previously reported for the R_2_Pd_3_Ge_5_ series.^15^

**Table 1:** Measured EDXS Compositions and Lattice Parameters Obtained from X-ray Studies

composition (atom %)			lattice parameters (Å)				
*R*	Pd	Ge	*a*	*b*	*c*	volume (Å^3^)	comment
La_2_Pd_3_Ge_5_							
20.2	30.2	49.6	10.183(2)	12.219(5)	6.1910(9)	770.3(3)	polycrystalline
			10.1914(6)	12.2082(7)	6.1901(4)	770.16(8)	single crystal
Nd_2_Pd_3_Ge_5_							
19.9	32.1	48.0	10.126(1)	12.061(2)	6.1203(4)	747.5(1)	polycrystalline
			10.1410(6)	12.0542(8)	6.1318(4)	749.56(8)	single crystal^15^

**Figure 1:**
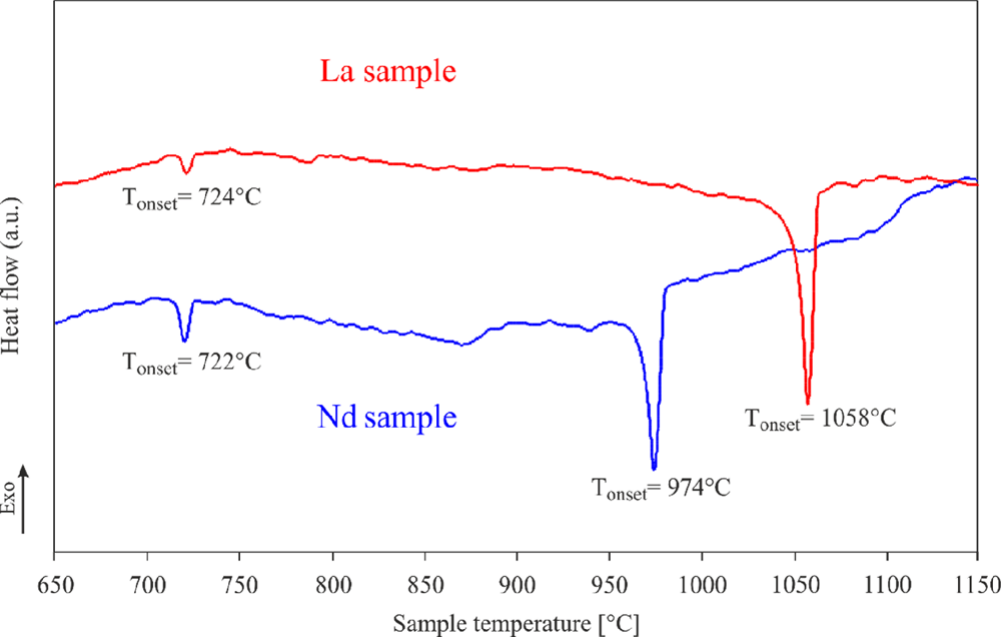
DTA heating curves of the La- and Nd-containing samples.

Two endothermic effects were recorded during DTA measurements, as shown in Figure 1. The studied compounds melt congruently at 1058 °C (La_2_Pd_3_Ge_5_) and 974 °C (Nd_2_Pd_3_Ge_5_). For both samples, the small peak at ∼725 °C indicates the melting of the (Ge+PdGe) eutectic mixture.^40^

### 3.1 Chemical Bonding

A quick glance at interatomic distances (see Table 2) confirms that, analogously to all known *R*_2_Pd_3_Ge_5_ intermetallics,^15^ also in La_2_Pd_3_Ge_5_ the Ge2 (8*g*) and Ge3 (8*j*) species are likely covalently bonded, forming infinite chains perpendicular to (001) planes, whereas Ge1 (4*a*) has no homocontacts. For the sake of simplicity, these species will be further labeled as two-bonded (2*b*) and zero-bonded (0*b*).

**Table 2:** La_2_Pd_3_Ge_5_ Interatomic Distances (< 3.6 Å) and Integrated Crystal Orbital Hamilton Populations (ICOHPs)

central atom	adjacent atom	*d* (Å)	*–*ICOHP (eV/bond)	central atom	adjacent atom	*d* (Å)	*–*ICOHP (eV/bond)
La (8*j*)	Ge3	3.1160(2)	1.03	Ge1 (4*a*)	Pd1(x4)	2.5446(1)	1.90
	Ge1(x2)	3.2558(1)	0.83	(0*b*)	Ge1(x2)	3.0951(2)	0.48
	Pd1	3.2559(1)	0.70		La(x4)	3.2558(1)	0.83
	Ge3(x2)	3.3129(2)	0.76		Ge2(x2)	3.3247(2)	0.19
	Ge3	3.3168(2)	0.78	Ge2 (8*g*)	Pd1(x2)	2.5097(1)	2.06
	Ge2(x2)	3.3399(1)	0.74	(2*b*)	Ge3(x2)	2.6348(1)	1.73
	Ge2(x2)	3.3411(1)	0.70		Pd2	2.7794(2)	1.18
	Pd1(x2)	3.3522(2)	0.62		Ge2(x2)	3.0951(2)	0.38
	Pd2(x2)	3.4928(1)	0.54		Ge1	3.3247(2)	0.19
	Pd1	3.5008(2)	0.44		La(x2)	3.3399(1)	0.74
	Pd1	3.5094(2)	0.47		La(x2)	3.3411(1)	0.70
Pd1 (8*j*)	Ge3	2.4635(1)	2.38	Ge3 (8*j*)	Pd1	2.4635(1)	2.38
	Ge2(x2)	2.5097(1)	2.06	(2*b*)	Pd2(x2)	2.5573(1)	1.94
	Ge1(x2)	2.5446(1)	1.90		Ge2(x2)	2.6348(1)	1.73
	La	3.2559(1)	0.70		La	3.1160(2)	1.03
	La(x2)	3.3522(2)	0.62		La(x2)	3.3129(2)	0.76
	La	3.5008(2)	0.44		La	3.3168(2)	0.78
	La	3.5094(2)	0.47				
Pd2 (4*b*)	Ge3(x4)	2.5573(1)	1.94				
	Ge2(x2)	2.7794(2)	1.18				
	Pd2(x2)	3.0951(2)	0.58				
	La(x4)	3.4928(1)	0.54				

According to the Zintl–Klemm approach, the presence of (2*b*)Ge and (0*b*)Ge could be accounted for by assuming a charge transfer from La and Pd to Ge, giving an ionic formula of (La^3+^)_2_(Pd^2+^)_3_[(2*b*)Ge^2–^]_4_[(0*b*)Ge^4–^]. Nevertheless, only a partial charge transfer is more probable considering the relatively small electronegativity difference between Ge and Pd referring to different scales (e.g., χ_Pd_ = 2.20, χ_Ge_ = 2.01, Pauling scale; χ_Pd_ = 4.45, χ_Ge_ = 4.60, Pearson absolute scale;^41^ χ_Pd_ = 1.58, χ_Ge_ = 1.994, Allen scale^42^).

**Figure 2:**
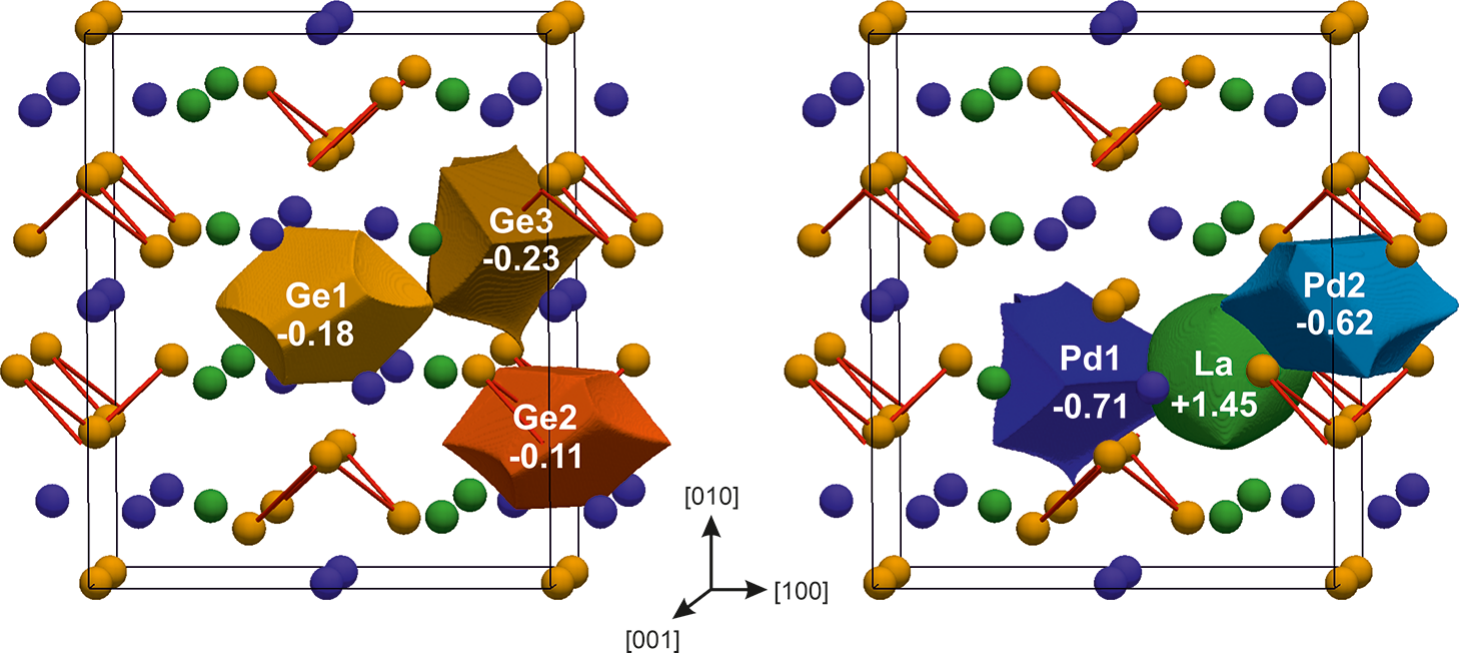
QTAIM basins for each Ge (left) and metal (La or Pd) species (right) together with the calculated atomic effective charges. Red sticks evidence the Ge–Ge contacts.

Instead, surprisingly, according to QTAIM effective charges (Figure 2), the only cationic species is La (+1.45). Nevertheless, its charge is considerably smaller than the formal one (+3), probably due to its participation in covalent bonds. A similar La behavior was recently described for La_2_*M*Ge_6_ compounds.^13^

The lowest negative charges are associated with Pd species: Pd2 (−0.62) is less negative than Pd1 (−0.71), which can be explained by the presence of Pd2–Pd2 contacts, with distances of ∼3.09 Å (see Table 2), whereas Pd1 is coordinated by only La and Ge. The anionic behavior of Pd and other noble metals, e.g., Pt, Au, and Ag, has been recently reported also for other rare-earth binary and ternary intermetallics.^13,20,43,44^ All of the Ge charges are close to zero, in contrast to the formal (2*b*)Ge^2–^ and (0*b*)Ge^4–^. The tiny negative charges indicate that interactions of Ge with the surrounding La and Pd should be interpreted as covalent rather than ionic. Additional information can be extracted from the shape of QTAIM atoms. In fact, La is rather spherical, which is typical for cations in similar compounds,^9,45−47^ and both Ge and Pd basins possess a polyhedral shape. What is more, not only the shared faces between Ge2 and Ge3 are flat, which is characteristic for homopolar bonds, but also those between Ge and Pd.

Hence, from the QTAIM effective charges, it turns out that the bonding scenario of La_2_Pd_3_Ge_5_ could not be adequately described by the simplified Zintl–Klemm scheme. This hypothesis is also borne out by the measured properties, presented in section 3.2, clearly indicating that this compound should not be classified as a Zintl phase.^48^

**Figure 3:**
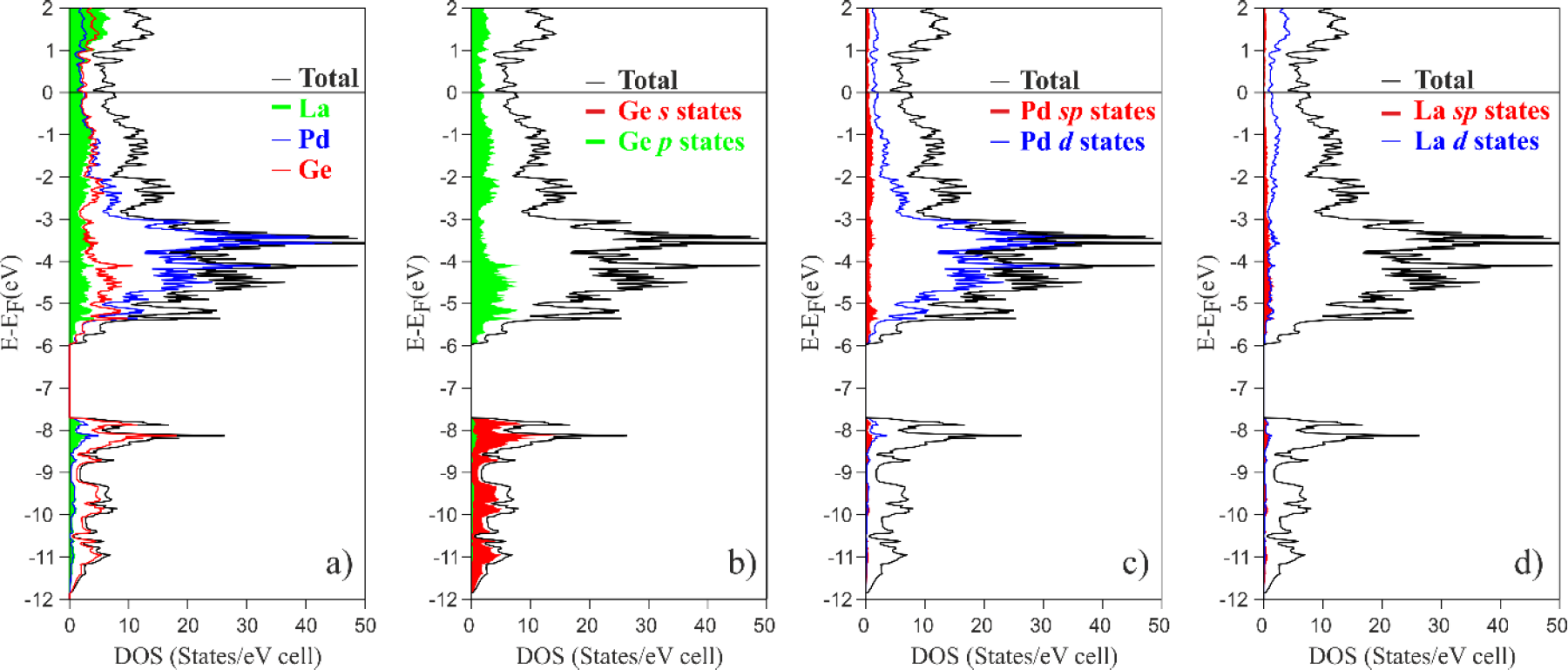
(a) Total density of states (DOS) for La_2_Pd_3_Ge_5_ together with species-projected (*p*DOS) and orbital *p*DOS for all (b) Ge, (c) Pd, and (d) La atoms.

These preliminary findings motivated us to give more insight into the chemical interactions among the constituents. Thus, the TB-LMTO-ASA method was applied and the obtained total and projected densities of states (DOS and *p*DOS, respectively) are shown in Figure 3.

La_2_Pd_3_Ge_5_ is a metal-like intermetallic, with a pseudogap at the Fermi level evidencing its electronic stability and overall bonding optimization. The lowest-energy region, below 7 eV, is mainly dominated by the Ge 4*s* states (Figure 3b), whereas the 4*p* states lie above, mixing considerably with Pd 4*d* (Figure 3c) and La 5*d* (Figure 3d), indicating the bonding nature of Ge–La/Pd interactions. The presence of the occupied La 5*d* states is a clear indication of its incomplete ionization due to the partial charge transfer, typical for rare-earth germanides. The region between approximately −6 and −2 eV is primarily dominated by Pd 4*d* states, showing a palladium behavior closer to that of a charge acceptor rather than of a typical cation, which is also confirmed by QTAIM effective charges.

**Figure 4:**
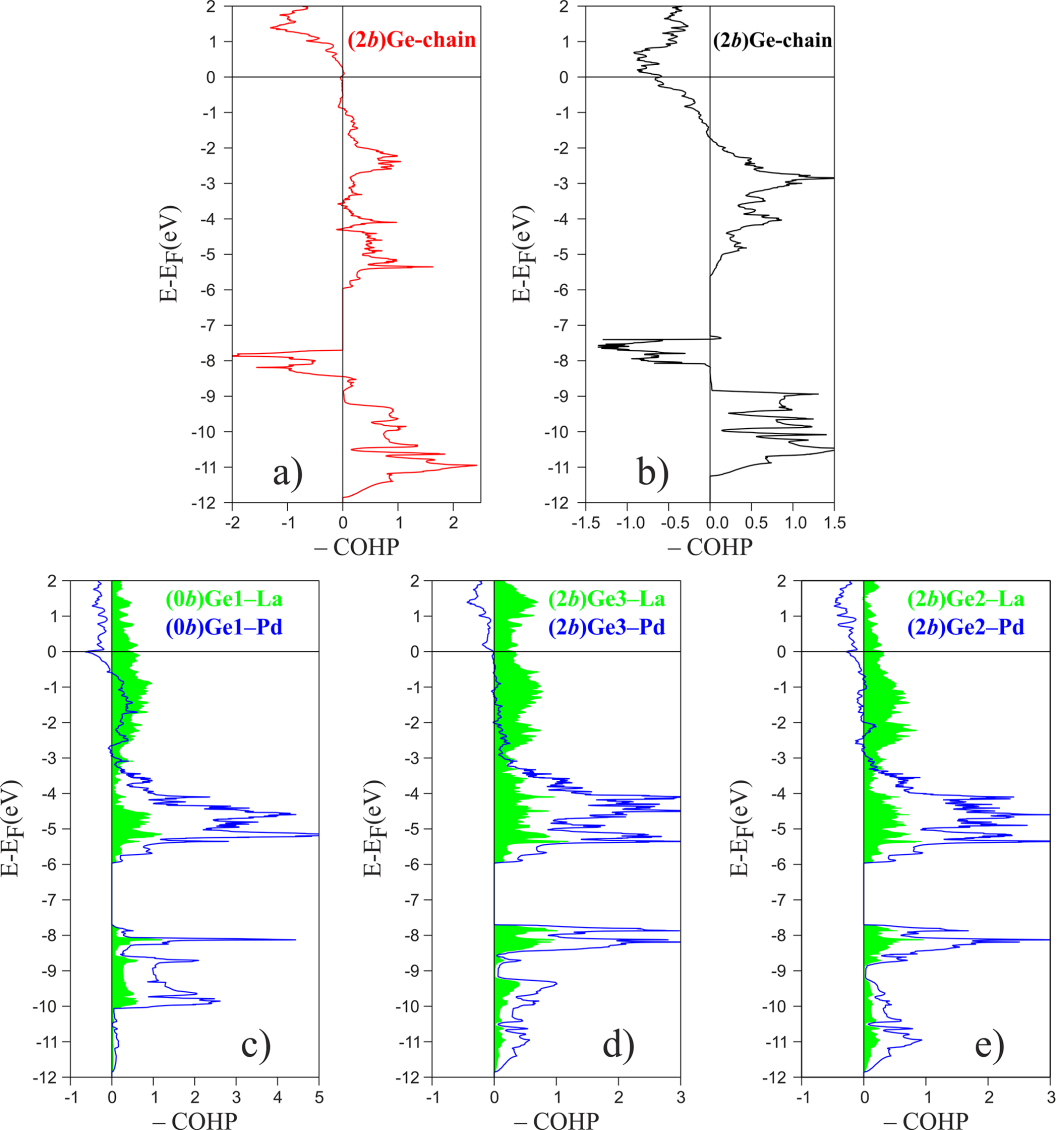
Crystal orbital Hamilton populations (−COHP curves) for selected Ge interactions in (a and c–e) La_2_Pd_3_Ge_5_ and (b) the hypothetical La_2_Mg_3_Ge_5_.

The presence of the (2*b*)Ge chains is coherent with the corresponding −COHP curve and −ICOHP value of 1.73 eV (see Figure 4a and Table 2, respectively). Interestingly, the −COHP curve is practically optimized at *E*_F_, revealing only a few antibonding states in the −1 to 0 eV energy window; in the case of anionic (2*b*)Ge^2–^ chains (with 2*e* lone pairs per atom according to the Lewis formula), one may expect more occupied antibonding states close to the Fermi level. The observed features suggest rather the existence of polar–covalent bonds between Ge species and the surrounding metals.

To corroborate this interpretation, a comparative chemical bonding analysis was conducted on the hypothetical “La_2_Mg_3_Ge_5_” compound, where the electropositive Mg was introduced in place of Pd. In fact, the −COHP plot for the (2*b*)Ge chains in the Mg-simulated analogue (Figure 4b) shows the presence of noticeable antibonding states below *E*_F_, approaching the Zintl description in terms of (2*b*)Ge^2–^ chains. This is also reflected in the reduced −ICOHP value of 1.29 eV (see the Supporting Information for all values) along with the increased Ge–Ge distance: 2.78 Å (La_2_Mg_3_Ge_5_) versus 2.63 Å (La_2_Pd_3_Ge_5_).

The Ge–metal bonding would be better depicted by the analysis of the corresponding −COHP curves (Figure 4 c-e). The Ge–Pd ones are weakly antibonding just below *E*_F_ in the case of Ge1/Ge2 and essentially nonbonding down to approximately −2 eV for Ge3. The Ge–Pd bonds are the strongest in the compound, ranging from 2.38 eV/bond for the shortest (2*b*)Ge3–Pd1 contact (2.46 Å) to 1.18 eV/bond for the (2*b*)Ge2–Pd2 contact (2.78 Å). The situation is different for the Ge–La interactions, which are weaker (1.03–0.70 eV) and not saturated, bonding also above *E*_F_.

With the aim of correctly discussing the magnitude of Ge–Pd and Ge–La ICOHP, the same values obtained for chemically related compounds may be used as references. For example, in GePd, *oP*8-FeAs,^49^ the average Ge–Pd distance is 2.56 Å, giving a −ICOHP of 1.76 eV. Upon comparison of these values with those for each Ge species in La_2_Pd_3_Ge_5_ (Ge1, 2.54 Å and 1.90 eV; Ge2, 2.60 Å and 1.77 eV; Ge3, 2.53 Å and 2.09 eV), the presence of strong polar covalent Ge–Pd bonds could be deduced. The same is also true for Ge–La interactions, which can be compared with analogous ones in La_2_ZnGe_6_, featuring very similar distances, COHP curves, and −ICOHP values.^11^ These findings have also been recently confirmed by position-space bonding analysis.^13^ In conclusion, because each Ge is bonded to the surrounding metals, which are always more numerous than the additionally realizable 2*c*–2*e* interactions (i.e., the number of lone pairs per Ge atom), the overall Ge–Pd/La bonding features a multicenter character.

**Figure 5:**
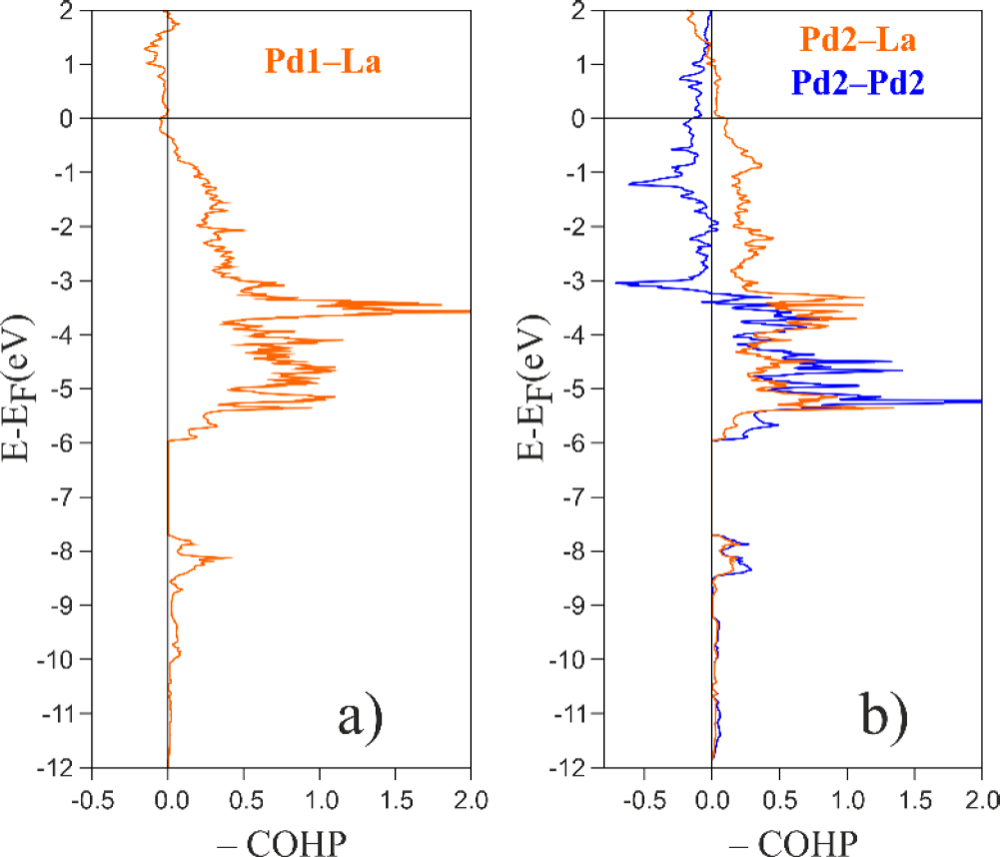
Crystal orbital Hamilton populations (−COHP curves) for Pd–La and Pd–Pd interactions in La_2_Pd_3_Ge_5_.

To complete the bonding analysis for La_2_Pd_3_Ge_5_, La–Pd and Pd–Pd bonds must also be taken into account. The Pd1 and Pd2 species are surrounded by five and four La atoms, respectively. As shown in panels a and b of Figure 5, all of the Pd–La interactions are optimized at *E*_F_, revealing their stabilizing role. The covalent nature of La–Pd interactions was also recently highlighted by means of the electron localizability indicator (ELI-D) and its “fine structure”, based on its relative Laplacian, in the LaPdGe_3_ and La_2_PdGe_6_ compounds,^13^ where Pd is coordinated by La in a topologically similar way to the title intermetallic.

Finally, Pd2–Pd2 contacts, with a distance of 3.09 Å, could be viewed as forming a one-dimensional linear chain, similar to that recently reported for Ca_2_Pd_2_Ge,^50^ where the Pd–Pd distance is 2.87 Å with a −ICOHP of 0.99 eV/bond. However, in La_2_Pd_3_Ge_5_, Pd atoms are remarkably less interactive, because their COHP curve (see Figure 5b) shows a noticeable number of occupied antibonding states starting from approximately −3 eV, leading to a −ICOHP of 0.58 eV/bond. Thus, in this case, the linear Pd2 chain should be mainly considered as a geometrical feature.

### 3.2 Physical Properties

**Figure 6:**
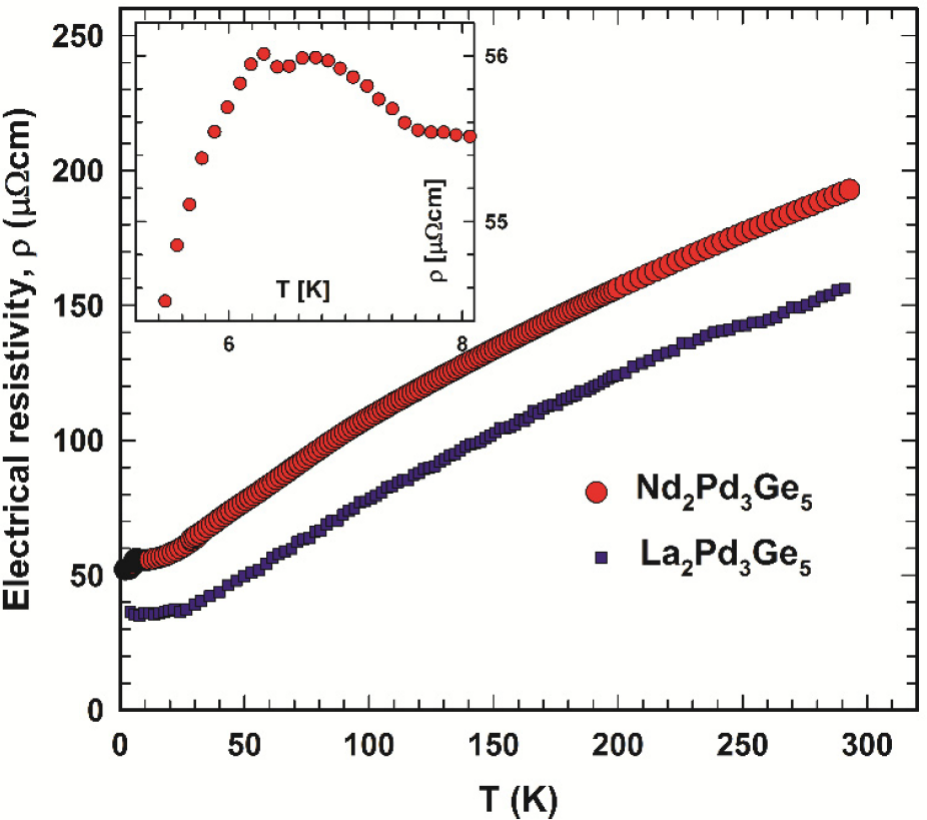
Temperature-dependent electrical resistivity ρ(*T*) of La_2_Pd_3_Ga_5_ and Nd_2_Pd_3_Ge_5_. The inset shows a low-temperature close-up of the ρ(*T*) anomalies of Nd_2_Pd_3_Ge_5_.

The temperature-dependent electrical resistivities, ρ(*T*), of La_2_Pd_3_Ge_5_ and Nd_2_Pd_3_Ge_5_ are displayed in Figure 6 and reveal the basically metallic nature of these compounds with a positive temperature coefficient.

**Figure 7:**
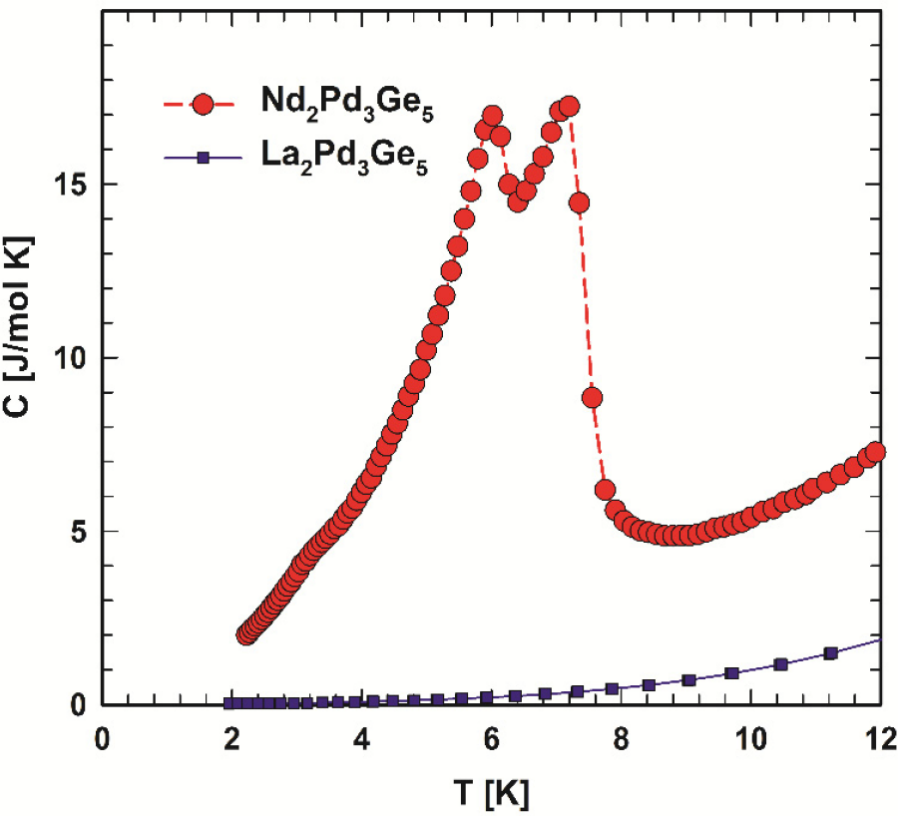
Temperature-dependent specific heat *C*_*p*_ of La_2_Pd_3_Ge_5_ and Nd_2_Pd_3_Ge_5_.

Their moderately large residual resistivity (ρ_0_ ∼ 40 and 50 μΩ cm, respectively) is likely to refer to some degree of disorder at the grain boundaries. The low-temperature resistivity of Nd_2_Pd_3_Ge_5_ increases to ∼6.3 K and then exhibits two anomalies: (i) a distinct drop followed by (ii) a weak increase, which typically results from an antiferromagnetic phase transition via superzone boundary scattering. The simple metallic behavior of La_2_Pd_3_Ge_5_ is well in line with the specific heat data (Figure 7), which at low temperatures (*T* < 6 K) follow the *C*(*T*) = γ*Τ* + β*Τ*^3^ function with an electronic Sommerfeld coefficient γ of 9.1(2) mJ mol^–1^ K^–2^ and a Debye temperature θ_D_ of 305(5) K of the lattice vibrations [β = 6.84(8) × 10^–4^ J mol^–1^ K^–4^]. The Sommerfeld coefficient extracted from the calculated DOS at *E*_F_ (compare Figure 3) (γ = 8.5 mJ mol^–1^ K^–2^) is in very good agreement with the experimental value.

**Figure 8:**
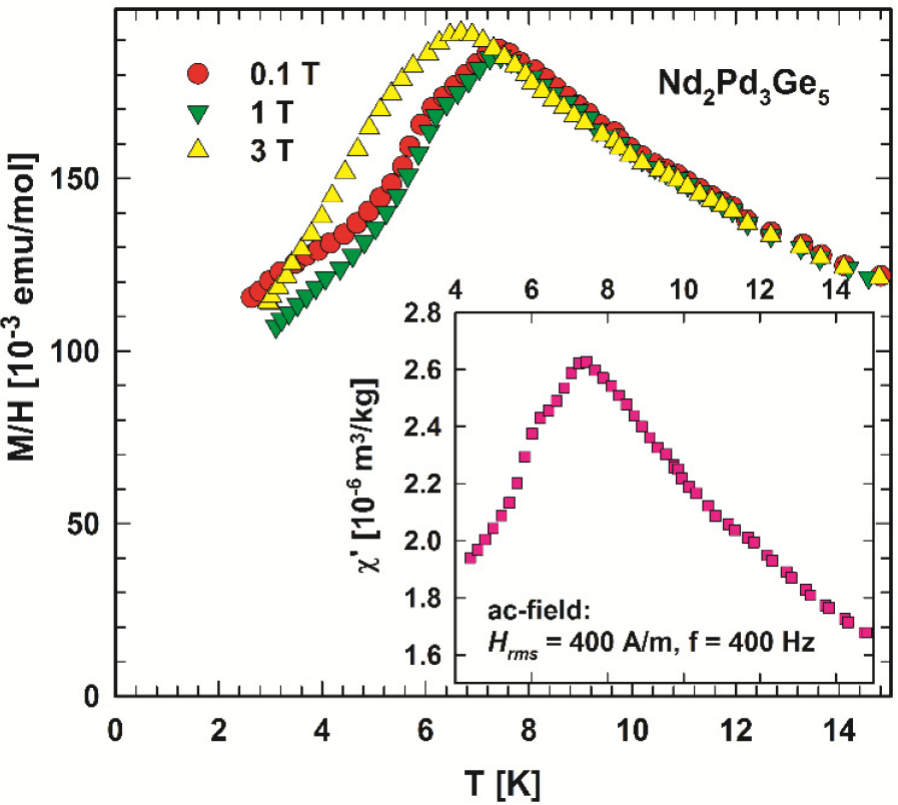
Temperature-dependent dc susceptibility, *M*/*H*, of Nd_2_Pd_3_Ge_5_ measured at field as labeled. The inset shows the in-phase component of ac susceptibility χ′ of Nd_2_Pd_3_Ge_5_.

The occurrence of two-phase transitions and the formation of an antiferromagnetic ground state of the Nd magnetic moments in Nd_2_Pd_3_Ge_5_ are confirmed by specific heat and magnetic susceptibility measurements. The specific heat of Nd_2_Pd_3_Ge_5_ (see Figure 7) displays two distinct phase transitions revealing the onset of long-range magnetic order at 7.5(3) K and a spin-reorientation transition at 6.2(2) K. The magnetic entropy gain, *S*_mag_(*T*) = *S*_Nd_(*T*) – *S*_La_(*T*), associated with the magnetic specific heat anomaly reaches *S*_mag_ = *R* ln 2 = 5.6 J mol^–1^ K^–1^ at 8.4 K, i.e., which conforms to the ordering of Nd moments in their crystalline electric field ground state doublet. The antiferromagnetic nature of the magnetic phase transition is confirmed by temperature-dependent ac and dc magnetic susceptibility data depicted in Figure 8. A distinct maximum of the in-phase component of the ac susceptibility χ′, as well as the 0.1 and 1 T dc susceptibility M/H, marks the Néel temperature at a *T*_N_ of 7.5 K (the out-of-phase ac susceptibility component χ″ ∼ 1.5 × 10^–7^ m^3^/kg remains essentially independent of temperature).

**Figure 9:**
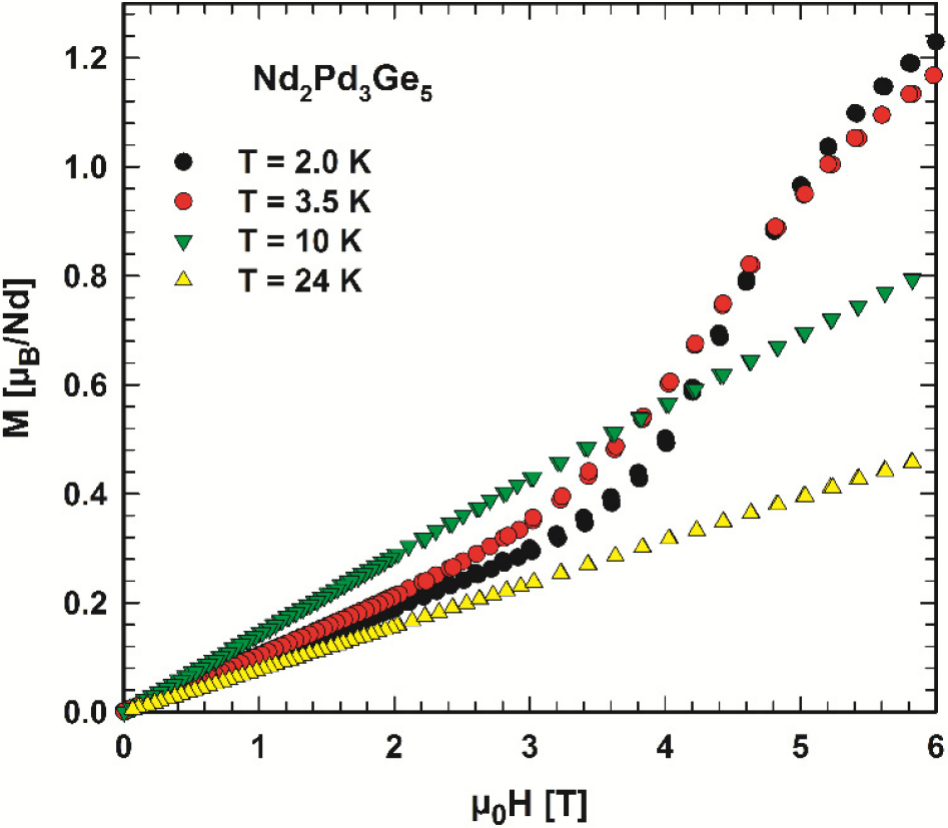
Magnetic isotherms, *M*(*H*), of Nd_2_Pd_3_Ga_5_ measured at various temperatures as labeled.

The spin-reorientation transition at 6.2 K relates to a distinct drop in the magnetic susceptibility when entering the magnetic ground state. At higher magnetic fields, as revealed by the 3 T dc magnetic susceptibility in Figure 8, the onset of antiferromagnetic order is shifted toward lower temperatures and the spin reorientation is suppressed. The latter is also confirmed by field-dependent specific heat data. The antiferromagnetic ground state is further supported by the typical spin-flop transition observed in the isothermal magnetization measurements at temperatures *T* < *T*_N_ and an applied magnetic field near 3.5–5.5 T (see Figure 9). The magnetization, measured at 2 K and a μ_0_*H* of 6 T, reaches 1.22 μ_B_/Nd, which is well below the theoretical free ion value (μ_sat_) of 3.27 μ_B_/Nd and refers to the specific crystalline electric field ground state of Nd_2_Pd_3_Ge_5_.

**Figure 10:**
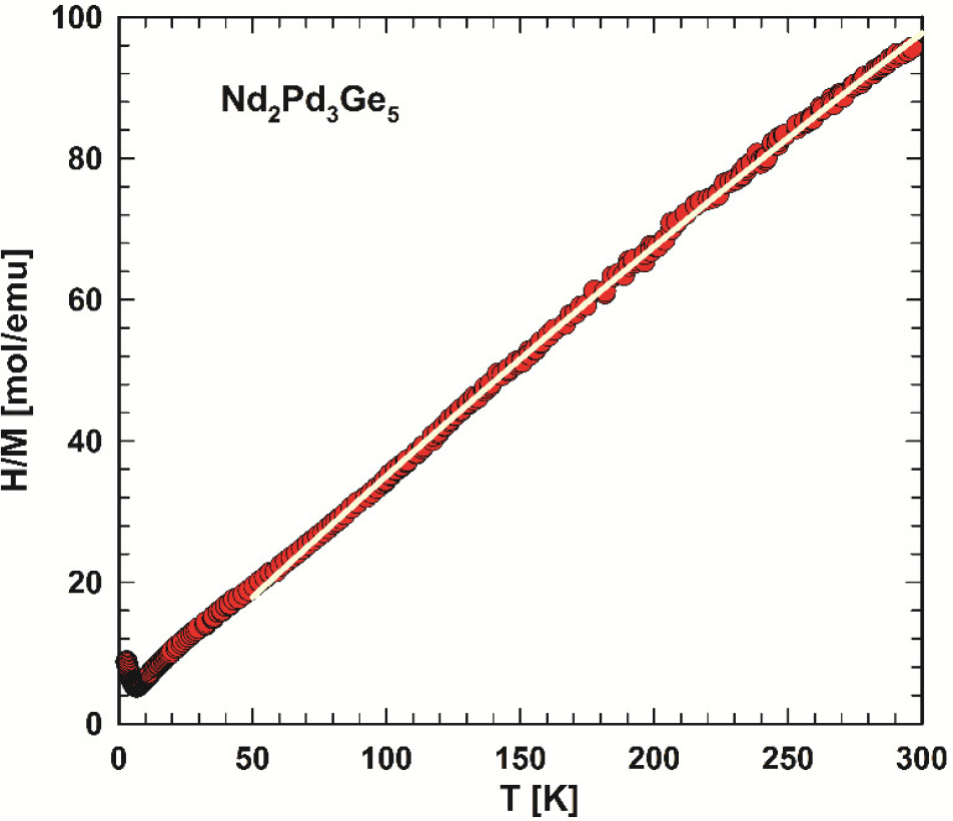
Temperature-dependent inverse dc magnetic susceptibility, *H*/*M*, of Nd_2_Pd_3_Ga_5_ measured in a magnetic field of 3 T. The solid white line indicates a modified Curie–Weiss fit (see the text).

Temperature- and field-dependent magnetization data of Nd_2_Pd_3_Ge_5_ in Figures 8 and 9 display a Curie–Weiss paramagnetic behavior above *T*_N_. To analyze the temperature dependence of the inverse susceptibility (3 T data), displayed in Figure 10 in the temperature interval from 50 to 300 K, a modified Curie–Weiss law, χ = χ_0_ + *C*/(*T* – θ_p_), was applied, where χ_0_ accounts for a temperature-independent Pauli susceptibility, θ_p_ is the paramagnetic Curie–Weiss temperature, and *C* is the Curie constant. The least-squares fitting yields a χ_0_ of 1.07 × 10^–3^ emu/mol, a θ_p_ of ≅0 K, and a Curie constant *C* of 2.75 emu K mol^–1^ and accordingly an effective magnetic moment μ_eff_ of 3.32 μ_B_/Nd, which is reasonably close to the theoretical free ion value of Nd^3+^, i.e., 3.62 μ_B_. The moderate reduction of the observed effective moment is attributed to the crystalline electric field splitting of the *J* = ^9^/_2_ multiplet of the Nd ions. The magnetic behavior of Nd_2_Pd_3_Ge_5_ is closely related to the even more complex magnetism as seen in a single-crystal study of Pr_2_Pd_3_Ge_5_,^51^ whereas isotypic Ce_2_Pd_3_Ge_5_ revealed simple antiferromagnetic order below a *T*_N_ of 3.8 K.^52^

## 4 Conclusions

The chemical bonding and physical properties of the two isotypic La_2_Pd_3_Ge_5_ and Nd_2_Pd_3_Ge_5_ intermetallic compounds (*oI*40-U_2_Co_3_Ge_5_) were investigated, enriching the available data for the numerous *R*_2_Pd_3_Ge_5_ series and enhancing our comprehension of their structure–bonding–property relationships.

Single-phase samples of the title compounds were obtained after arc melting. Differential thermal analysis measurements indicate that they melt congruently at 1058 °C (La_2_Pd_3_Ge_5_) and 974 °C (Nd_2_Pd_3_Ge_5_). Refined structural data for the La-containing analogue, essential for the chemical bonding investigations, were obtained by single-crystal X-ray diffraction analysis.

Analysis of QTAIM effective charges, DOS and −COHP curves, and −ICOHP values revealed that, in addition to covalent Ge–Ge bonds forming infinite zigzag chains, polar Ge–Pd/La multicenter interactions occur, resulting from the incomplete charge transfer to Ge. The bonding scenario is further complicated by the fact that Pd and La are also covalently interacting.

The findings of chemical bonding studies are consistent with the measurements of physical properties: in fact, electrical resistivity as a function of temperature shows that both compounds are metallic. While La_2_Pd_3_Ge_5_ exhibits a simple metallic behavior, the resistivity trend of Nd_2_Pd_3_Ge_5_ indicates two low-temperature phase transitions, in accordance with specific heat and susceptibility measurements. An antiferromagnetic ground state is established after a long-range magnetic ordering (at ∼7.5 K) followed by a spin-reorientation transition (at ∼6.2 K).

## Supporting Information Available

Crystallographic data for the La_2_Pd_3_Ge_5_ single crystal (Tables S1 and S2), experimental and relaxed unit cell parameters for the existing La_2_Pd_3_Ge_5_ and simulated La_2_Mg_3_Ge_5_ compounds (Table S3), interatomic distances and −ICOHP values for simulated La_2_Mg_3_Ge_5_ (Table S4), additional details about the calculations performed for La_2_Mg_3_Ge_5_, and SEM images and X-ray powder patterns of the measured samples (Figures S1–S3) (PDF)

## Accession Codes

CCDC 1917376 contains the supplementary crystallographic data for this paper. These data can be obtained free of charge via www.ccdc.cam.ac.uk/data_request/cif, or by emailing data_request@ccdc.cam.ac.uk, or by contacting The Cambridge Crystallographic Data Centre, 12 Union Road, Cambridge CB2 1EZ, UK; fax: +44 1223 336033.
