# Supplementary material for: La2Pd3Ge5 and Nd2Pd3Ge5 Compounds: Chemical Bonding and Physical Properties
Source: Inorg Chem. 2021 Feb 11;60(5):3345–54. doi: 10.1021/acs.inorgchem.0c03744 (PMC8023660; doi:10.1021/acs.inorgchem.0c03744)
Supplement: Supplementary file 1 — ic0c03744_si_001.pdf [file ic0c03744_si_001.pdf]

$La_2Pd_3Ge_5$ and $Nd_2Pd_3Ge_5$ Compounds: Chemical Bonding and Physical Properties

Riccardo Freccero[1]*, Serena De Negri[1], Gerda Rogl[3], Georg Binder[4], Herwig Michor[4], Peter F. Rogl[3], Adriana Saccone[1], Pavlo Solokha[1]

[1]*Università degli Studi di Genova, Dipartimento di Chimica e Chimica Industriale, Via Dodecaneso 31, I-16146 Genova, Italy*

[3]*Institute of Materials Chemistry, University of Vienna, Währingerstraße 42, A-1090 Vienna, Austria*

[4]*Institute of Solid State Physics, TU Wien, Wiedner Hauptstraße, 8-10, A-1040, Wien, Austria*

Supporting Information

* To whom the correspondence should be addressed: Riccardo Freccero; E-mail: riccardo.freccero@edu.unige.it

Table S1. Crystallographic data for $La_2Pd_3Ge_5$ single crystal and selected experimental details of the structure solution.

Empirical formula	**$La_2Pd_3Ge_5$**
EDXS data	$La_{20.7}Pd_{32.2}Ge_{47.3}$
Lattice parameters [Å]	*a* = 10.1914(6); *b* = 12.2082(7); *c* = 6.1901(4)
V [Å^3]	770.16(8)
Calc. density [g/cm^3]	8.279
Abs. coeff. (μ), mm^{-1}	36.8
Unique reflections	676
Reflections I > 2σ(I); R_{sigma}	662; 0.0062
Data/parameters	662/50
GOF on F^2 (S)	1.019
R indices [I > 2σ(I)]	R1 = 0.0092; wR2 = 0.0320
R indices [all data]	R1 = 0.0098; wR2 = 0.0322
$\Delta\rho_{fin}$ (max/min), [e/Å^3]	0.705/-0.680

Table S2. Standardized atomic coordinates and equivalent isotropic displacement parameters for $La_2Pd_3Ge_5$.

Atom	*Site*	*x/a*	*y/b*	*z/c*	U_{iso} (Å^2)
La	8*j*	0.26540(2)	0.37077(2)	0	0.00589(7)
Pd1	8*j*	0.10873(2)	0.13834(2)	0	0.00715(7)
Pd2	4*b*	0.5	0	0.25	0.00873(9)
Ge1	4*a*	0	0	0.25	0.00707(1)
Ge2	8*g*	0	0.27233(3)	0.25	0.00726(8)
Ge3	8*j*	0.34769(3)	0.10791(3)	0	0.00737(8)

Table S3. Experimental and relaxed unit cell parameters for the existing $La_2Pd_3Ge_5$ and the simulated $La_2Mg_3Ge_5$ compounds.

	**Experimental**				**Calculated**			
Compound	*a* (Å)	*b* (Å)	*c* (Å)	V (Å^3)	*a* (Å)	*b* (Å)	*c* (Å)	V (Å^3)
$La_2Pd_3Ge_5$	10.1914(6)	12.2082(7)	6.1901(4)	770.16(8)	10.1021	12.0363	6.1437	747.03
$La_2Mg_3Ge_5$	-	-	-	-	10.7112	13.0326	6.1713	861.47

Table S4. Interatomic distances (< 3.95 Å) and Integrated, up to the E_F, Crystal Orbital Hamilton Population (ICOHP) for the simulated $La_2Mg_3Ge_5$.

Central atom	Adjacent atoms	*d* (Å)	–ICOHP (*eV/bond*)	Central atom	Adjacent atoms	*d* (Å)	–ICOHP (*eV/bond*)
**La**	Ge3	3.2098	1.16	**Mg2**	Ge2(x2)	2.7028	1.28
	Ge3(x2)	3.2320	1.03		Ge3(x4)	2.8096	1.20
	Ge2(x2)	3.3541	0.83		Mg2(x2)	3.0856	0.51
	Ge1(x2)	3.4335	0.89		La(x4)	3.6288	0.35
	Mg1	3.4707	0.32	**(0*b*)Ge1**	Mg1(x4)	2.5804	1.61
	Mg1(x2)	3.4930	0.37		Ge1(x2)	3.0856	0.51
	Ge3	3.4960	0.74		La(x4)	3.4335	0.89
	Mg1	3.5082	0.37		Ge2(x2)	3.8135	-0.05
	Mg2(x2)	3.6288	0.35	**(2*b*)Ge2**	Mg1(x2)	2.6584	1.36
	Ge2(x2)	3.6495	0.50		Mg2	2.7028	1.28
	Mg1	3.9151	0.16		Ge3(x2)	2.7877	1.29
**Mg1**	Ge3	2.5779	1.66		Ge2(x2)	3.0856	0.51
	Ge1(x2)	2.5804	1.61		La(x2)	3.3541	0.83
	Ge2(x2)	2.6584	1.36		La(x2)	3.6495	0.50
	La	3.4707	0.32		Ge1	3.8135	-0.05
	La(x2)	3.4930	0.37	**(2*b*)Ge3**	Mg1	2.5779	1.66
	La	3.5082	0.37		Ge2(x2)	2.7877	1.29
	Mg1	3.5911	0.05		Mg2(x2)	2.8096	1.20
	La	3.9151	0.16		La	3.2098	1.16
					La(x2)	3.2320	1.03
					La	3.4960	0.74

Additional details about the calculations performed for $La_2Mg_3Ge_5$

No empty spheres were added. The following radii of the atomic spheres were applied for the calculation: *r*(La) = 2.201 Å, *r*(Ge1) = 1.572 Å, *r*(Ge2) = 1.622 Å, *r*(Ge3) = 1.569 Å, *r*(Mg1) = 1.459 Å and *r*(Mg2) = 1.552 Å. The self-consistent calculation was performed with a basis set including La-6*s*/(6*p*)/5*d*/4*f*, Ge-4*s*/4*p*/(4*d*) and Mg-3*s*/3*p*/(3*d*); parentheses indicate orbitals treated according to a downfolding procedure.

Since symmetry is not included in FHI-aims calculations, the relaxed structure was set again to the *Ibam* space group, in order to perform the comparative analysis with the Pd analogue.

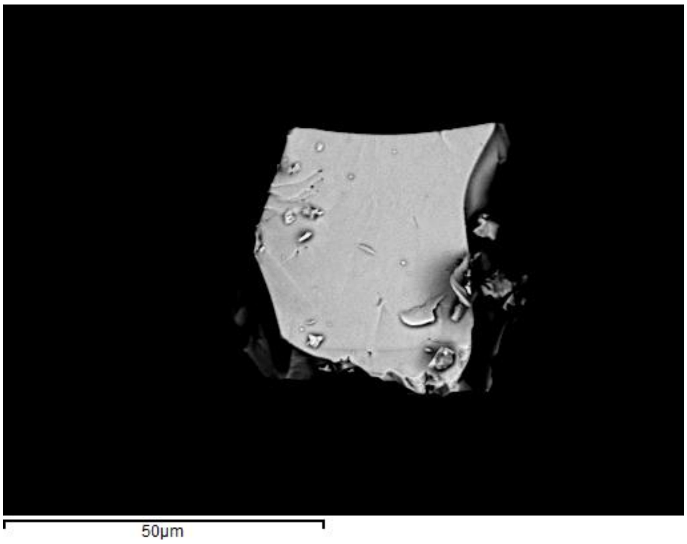

Figure S1. SEM image (BSE mode) of the $La_2Pd_3Ge_5$ single crystal analysed by X-ray diffraction.

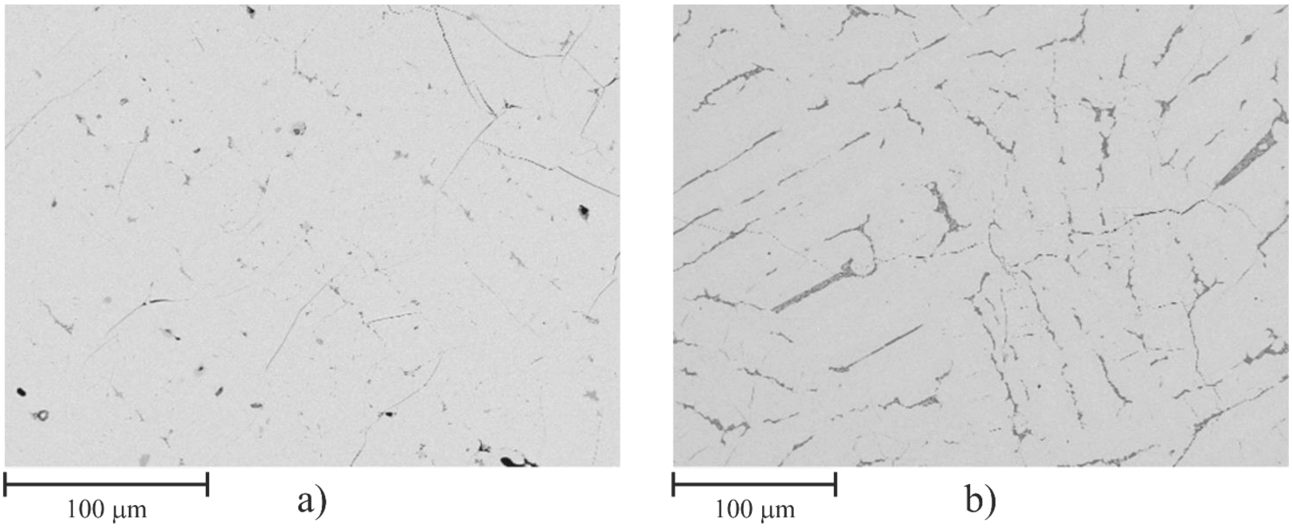

Figure S2. SEM micrographs (BSE mode) of a) La sample and b) Nd sample. The main phase is $R_2Pd_3Ge_5$; at the grain boundary a small amount of the eutectic Ge+PdGe mixture is present.

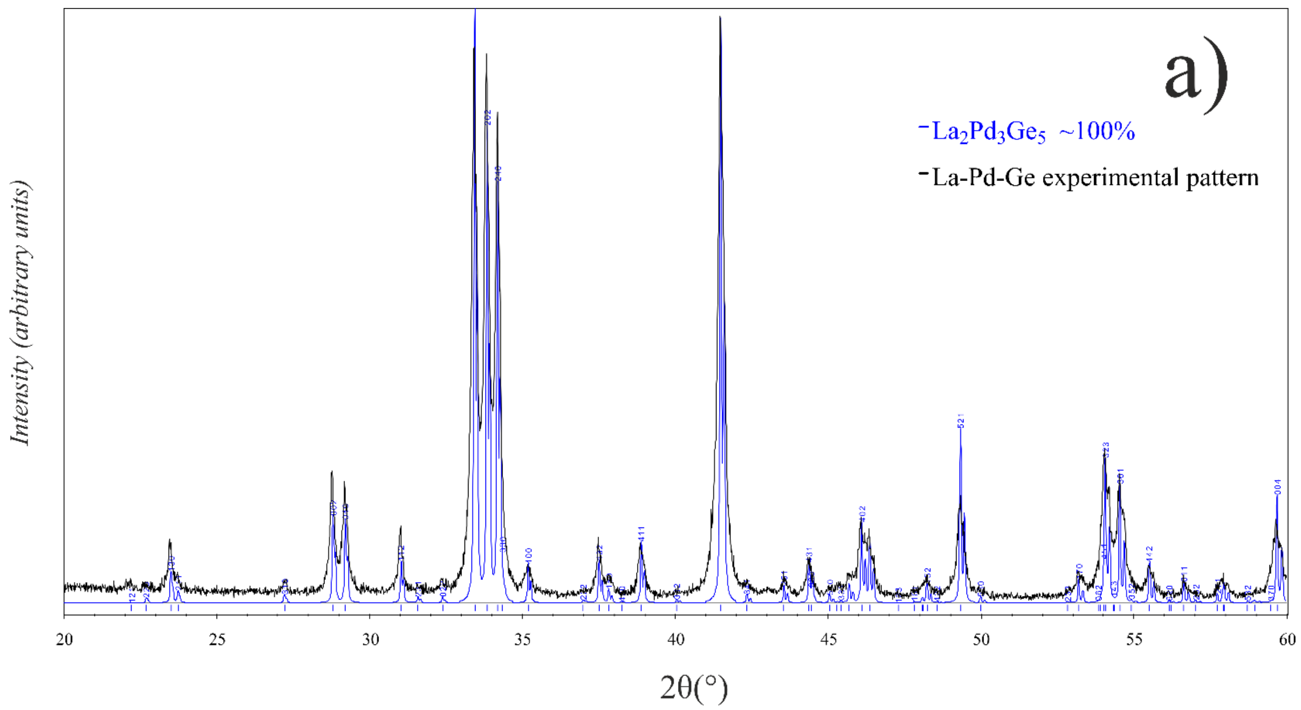

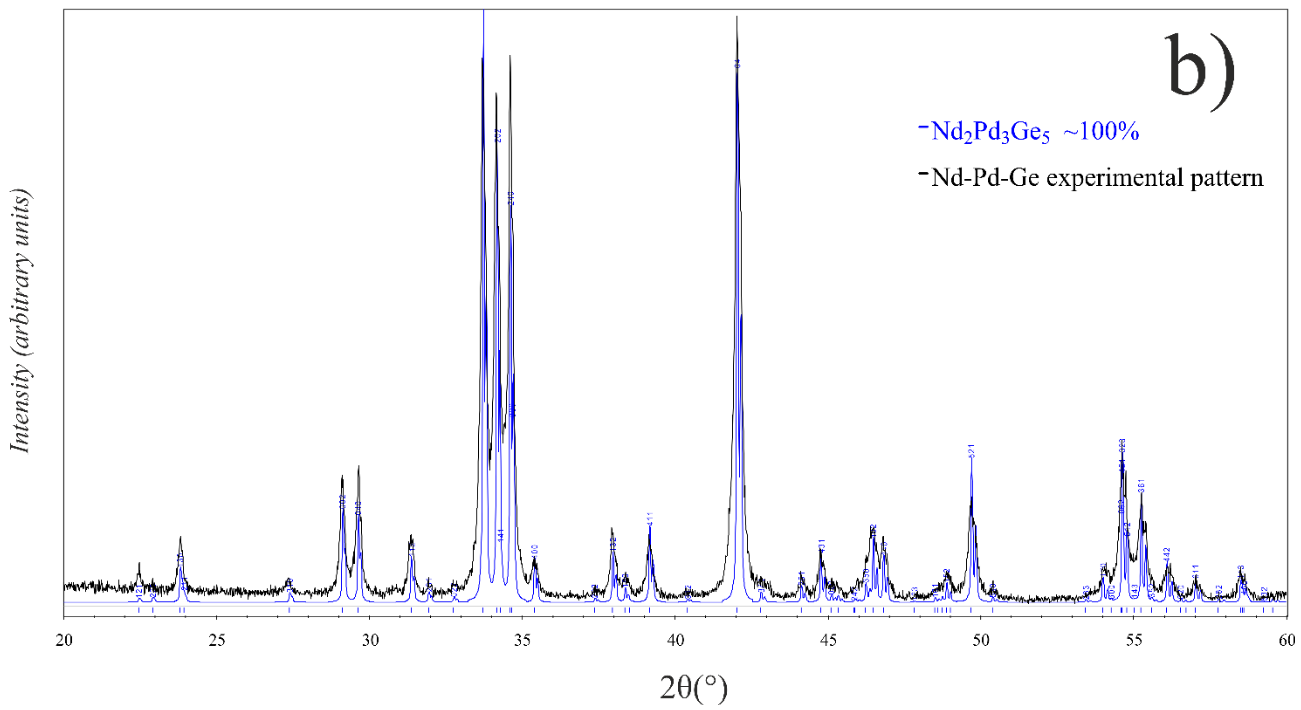

Figure S3. X-ray powder patterns of the almost single-phase a) La and b) Nd samples.
